# Meniscal Allograft Transplantation Concomitant With Cartilage Repair for Symptomatic Lateral Meniscus-Deficient Knees With Over Two Years of Follow-up

**DOI:** 10.7759/cureus.48774

**Published:** 2023-11-14

**Authors:** Yuji Uchio, Hiroshi Takuwa, Takuya Wakatsuki, Suguru Kuwata

**Affiliations:** 1 Department of Orthopaedic Surgery, Shimane University, Izumo, JPN; 2 Department of Orthopaedic Surgery, National Hospital Organization Hamada Medical Center, Hamada, JPN

**Keywords:** lateral meniscus injury, osteochondral transplantation, autologous chondrocyte transplantation, chondrocyte transplantation, meniscal allograft, meniscus-deficient knee

## Abstract

Background and objective

The treatment for symptomatic meniscus-deficient knees with cartilage defects remains challenging on account of insufficient meniscal substitutes. One solution for this might involve combining meniscal allograft transplantation (MAT) and cartilage repair. In this study, we aimed to analyze the effectiveness and safety of MAT concomitant with cartilage repair for symptomatic lateral meniscus-deficient knees in a setting with limited availability of meniscal transplants in Japan.

Methods

Nine patients who underwent MAT concomitant with osteochondral transplantation (five) and/or autologous chondrocyte implantations (seven) were followed up for at least two years (mean: 51.2 months, range: 24-84 months). Their demographic data and other characteristics were as follows - mean age: 51.7 years, range: 36-67 years; men/women: 4/5; cause: trauma/discoid meniscus: 8/1; cartilage defect size: mean: 6.7 cm^2^/knee, range: 1.0-11.3. The effectiveness and safety were evaluated clinically by using the Lysholm Knee Scoring Scale (LKSS) and Japanese Orthopaedic Association (JOA) knee score, physical examination, X-rays, and MRI preoperatively and at one, 12, and 24 months after the implantation. Differences between the variables were analyzed using the Friedman test and Scheffe's multiple comparisons.

Results

The median LKSS and JOA scores significantly improved from 70 points (range: 21-80) and 35 (25-45) preoperatively to 86.5 (65-98) and 87.5 (80-95) at 24 months after surgery, respectively (p<0.001, p=0.0013). The range of motion (ROM), femorotibial angle, and the lateral joint space showed no significant changes. However, lateral meniscal extrusions (LMEs) increased by 3.0 mm (range: 0-6.3 mm) at one month postoperatively and remained unchanged until two years postoperatively. Treatment failure occurred in one case, which was revised by total knee arthroplasty (TKA) at 18 months postoperatively. Additional surgeries were needed in some cases: lateral meniscal tear (three cases), contracture (two cases), and patellar instability (one case). However, neither infection nor allergic reaction was observed in the blood exams.

Conclusions

Although MAT concomitant with cartilage repair showed good clinical outcomes, half of the cases needed additional surgeries. Based on our findings, this technique should be adopted only in select cases and performed by a handful of highly experienced surgeons.

## Introduction

Meniscal tear is a common orthopedic injury, and it is usually treated by meniscectomy [1] to relieve symptoms, such as pain, clicking, or locking, and improve activities of daily living. Although symptoms tend to improve promptly after meniscectomy, several retrospective studies employing X-rays and MRI have reported a high incidence (33-66%) of cartilage wear and degeneration, subchondral bone lesion, and joint narrowing after meniscectomy during long-term follow-up [2-4], revealing the risks of osteoarthritis (OA) in the meniscectomized knee [5]. A prospective study demonstrated that radiographic OA occurred in 40% of athletes at 4.5 years following meniscectomy, which rose to 89% at 14.5 years [6]. A systematic review has demonstrated a higher incidence of OA in total meniscectomy than in partial meniscectomy, indicating the importance of saving the meniscus [7]. Despite the availability of many techniques to preserve the critical biomechanical properties of load distribution and stability, ranging from meniscectomy to meniscal repair [1], a definitive method for saving the meniscus has yet to be devised owing to the lack of appropriate meniscal substitutes [8,9].

Meniscal allograft transplantation (MAT) might aid in restoring native biomechanics and stability to the meniscus-deficient knee so that rapid OA degeneration can be delayed or prevented. Since it was first described by Milachowski et al. in 1989 [10], MAT has been considered an alternative treatment option for meniscectomized knees in young patients [11,12]. A review of 55 studies demonstrated that MAT could yield good clinical results in the short and medium terms, with improved knee function and acceptable complication and failure rates [13]. Except for three cases, this review excluded patients with combined Outerbridge grade III and grade IV cartilage damages [13]. In 2002, Gersoff reported 19 cases of meniscus-deficient knees and an average cartilage defect of 8.4 cm^2^, which were treated with a combination of MAT and autologous chondrocyte transplantation (ACI), resulting in improved knee function with minimal complication at an average follow-up duration of 24.7 months [14]. In 2016, Ogura et al. described MAT concomitant with ACI in 18 symptomatic meniscus-deficient knees with cartilage damage, leading to significant improvement in 65% of the knees and a 75% survival rate at five and 10 years [15].

In contrast, no case of MAT concomitant with articular surgery has ever been reported in Japan. Since the first heart transplantation in 1968, which drew significant socio-medical criticism, no allograft from donors after brain and cardiac death had been legally permitted in Japan until the Law concerning Human Organ Transplants took effect in 1997 [16]. Between 1999 and 2016, organs from 796 brain-dead donors and 1,732 heart-dead individuals were used for 5,623 transplantations involving hearts, lungs, livers, kidneys, pancreas, and small intestines [16]. However, despite the fact that several meniscectomies have been performed in Japan, meniscal allografts, even from heart-dead donors, have not been applied to symptomatic meniscus-deficient knees. Since 2016, the cost of meniscus allografts with bone from tissue banks authorized by the Japanese Society of Tissue Transplantation has been covered by the universal healthcare system of Japan. Accordingly, this universal healthcare system initiated the provision of MAT with cartilage repair for symptomatic meniscus-deficient knees with cartilage defects. This case series pilot study examines the effectiveness and safety of MATs combined with osteochondral autograft/allograft transplantation (OAT) and/or ACI for symptomatic lateral meniscus-deficient knees with cartilage defects with at least two years of follow-up, including previously published case reports [17,18].

## Materials and methods

Ethical considerations

The MAT protocol was approved by the IRB of the authors' affiliated institutions. The Tokai Regional Tissue Bank (Nagoya, Japan) and Aichi Bone Soft Tissue Transplantation Foundation (Nagoya, Japan) provided the supply of fresh-frozen meniscus with tibial plateau bone from heart-dead donors to the authors' affiliated institutions under the Japanese universal healthcare system, the permission for which was granted by the Regional Bureau of Health and Welfare in Japan. All patients who participated in this study provided informed consent.

Inclusion and exclusion criteria

This study included patients treated at our hospital with symptomatic meniscus-deficient knee combined with articular cartilage defects despite undergoing conservative treatments such as medication, physical therapy, and bracing over three months. All patients were aged over 20 years and less than 70 years. Patients who were treated for rheumatoid arthritis or infectious diseases were excluded. Previously reported cases [17,18] considered in this study had the same study design and patients in those studies went through the same procedures and protocols as the participants in the current study.

Patient demographics

The mean age of the patients was 51.7 years (range: 36-67) and the mean body mass index was 24.0 kg/cm^2^ (range: 17.2-30.2); four patients were men, and five were women. The causes of the meniscus-deficient knee with articular defects were as follows: trauma (eight knees) and post-meniscectomy due to discoid meniscus (one knee). The mean cartilage defect lesion size was 6.7 cm^2^/knee (1.0-11.3). Articular cartilage surgeries concomitant with MAT included OAT (four/one) and/or ACIs (seven). In two cases, MAT was combined with anterior cruciate reconstruction (ACL-R), one of the two with medial meniscal suture. The mean follow-up period was 46.2 months (range: 24-76 months). The patient details are summarized in Tables 1-2.

**Table 1:** Demographic data of the patients (1) M: male; F: female; L: left; R: right; SD: standard deviation; SE: standard error; ACL-R: anterior cruciate ligament reconstruction; LDM: lateral discoid meniscus; LM: lateral meniscus

No.	Age (years)	Gender	Laterality	Height (cm)	Weight (kg)	BMI (kg/m^2^)	Causes	Previous surgeries
									1	52	F	L	165	47	17.2	Trauma	ACL-R, LM suture
									2	42	M	L	175	67	21.8	Trauma	None
									3	36	F	L	165	65.9	24.4	LDM	LDM meniscectomy
4	47	F	R	162	68.6	26.3	Trauma	None
5	46	M	R	171	76.6	26.1	Trauma	None
6	56	F	R	160	61.2	24	Trauma	None
7	67	M	L	175	80.8	26.4	Trauma	None
8	55	F	L	166	54.7	19.9	Trauma	ACL-R, LM suture
9	64	M	R	161	78.3	30.2	Trauma	LM meniscectomy
Mean	51.7			166.6	66.7	24		
SD	10.1			5.9	11.2	3.9		
SE	3.4			2	3.7	1.3		

**Table 2:** Demographic data of the patients (2) SD: standard deviation; SE: standard error; LFC: lateral femoral condyle; LTP: lateral tibial plateau; FT: femoral trochlear; P: patella, MFC: medial femoral condyle; ACI: autologous chondrocyte transplantation; OAT: osteochondral autograft/allograft transplantation; ACL-R: anterior cruciate ligament reconstruction; LM: lateral meniscus; MPFL-R: medial patellofemoral ligament reconstruction; ATT: anteromedial transfer of the tibial tuberosity; TKA: total knee arthroplasty

No.	Cartilage defect		Cartilage repair	Combined surgeries	Additional surgeries	Follow-up (months)
	Size (cm^2^)	Location				
1	4.0	LFC, LTP	ACI (LFC), OAT (LTP)		LM suture	76
2	1.0	LFC	OAT (LFC)	ACL-R + MM suture	LM suture	73
3	9.3	LFC, P	ACI (LFC, P) + OAT (LTP)		Athrolysis + MPFL-R + ATT + LM suture	60
4	11.3	MFC, LFC, P	ACI (MFC) + OAT (LFC, P)	ACL-R	Athrolysis	48
5	7.5	LFC, LTP	ACI (LFC)			37
6	10.3	LFC, LTP, FT	ACI (LFC, FT)			36
7	10.0	LFC, LTP	ACI (LFC)			36
8	6.0	LFC, LTP, FT	ACI (LFC, FT)		TKA	26
9	1.0	LTP	OAT (LTP)			24
Mean	6.7					46.2
SD	3.9					19.3
SE	1.3					6.4

Preoperatively, patients' CT images and MRI were brought to the Tokai Regional Tissue Bank to match the size of the meniscal allografts. The meniscus size data for both length and width measurements between donors and patients are shown in Table 3.

**Table 3:** Meniscal size of patients and allografts Mann-Whitney U test showed no significant meniscal length and width differences between patients and allografts SD: standard deviation; SE: standard error

No.	Patient		Allograft	
	Length (mm)	Width (mm)	Length (mm)	Width (mm)
1	37	36	37	35
2	39	33	39	32
3	33	30	33	31
4	33	31	33	31
5	35	31	35	28
6	38	32	35	32
7	38	30	40	28
8	37	30	35	30
9	37	31	35	28
Mean	36.3	31.6	35.8	30.6
SD	2.2	1.9	2.4	2.4
SE	0.7	0.6	0.8	0.8

Surgical procedures

One senior surgeon who had performed more than 3300 orthopedic surgeries (1289 cruciate ligament reconstructions, 478 meniscectomies, 343 meniscal sutures, 492 OATs, 49 ACIs, 169 around knee osteotomies, etc.) performed all the procedures.

MAT

**Figure 1:**
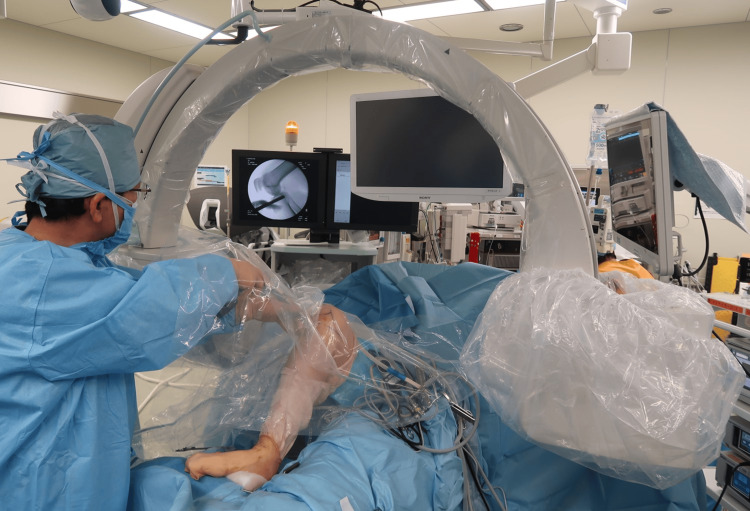
The creation of the trapezoidal slot for meniscal allograft transplantation under fluoroscopy

Under general anesthesia, intra-articular structures were evaluated via arthroscopy. Medial meniscal repair and ACL-R were performed as needed. Following this, a deep-frozen meniscal allograft with tibial plateau bone supplied by the Tokai Regional Tissue Bank (Nagoya, Japan) was prepared on the operation table. By modifying Dovetail's method [19] via anterolateral arthrotomy, a trapezoidal slot (articular surface upper side; 8 mm, a lower side; 10 mm, depth; 10 mm, length; 45-50 mm) was created at the lateral tibial plateau (LTP) near the lateral intercondylar eminence without penetrating the posterior tibial cortex by using a core reamer, chisel, and custom-made sizer under arthroscopy and fluoroscopy (Figure 1).

**Figure 2:**
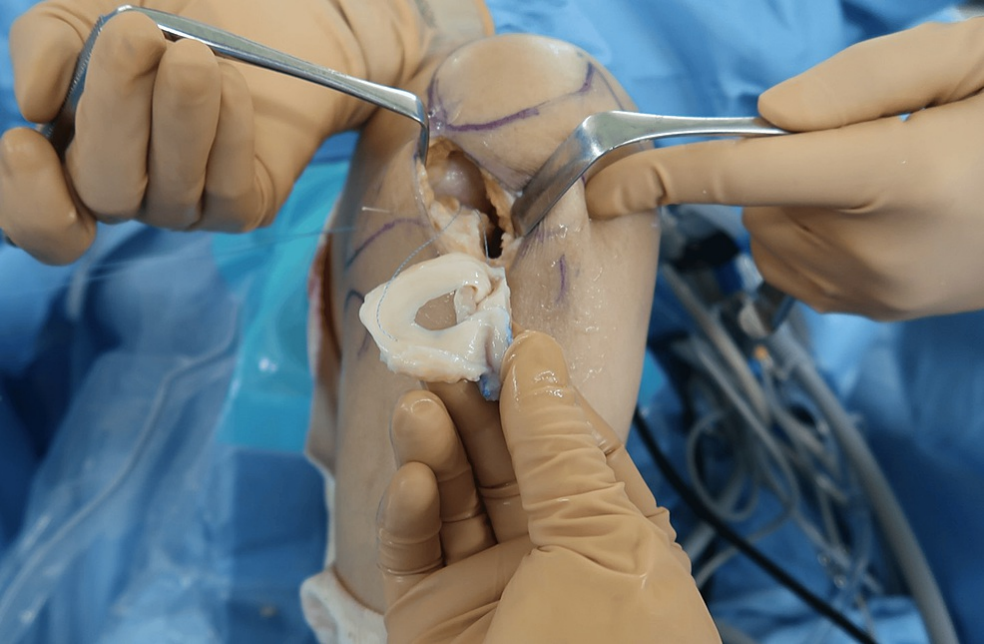
The adaptation of the bone bridge with allogenic meniscus into the slot

A shaped bone bridge with a meniscal allograft was inserted into the slot via an anterolateral arthrotomy to the lateral femorotibial (FT) compartment (Figure 2).

**Figure 3:**
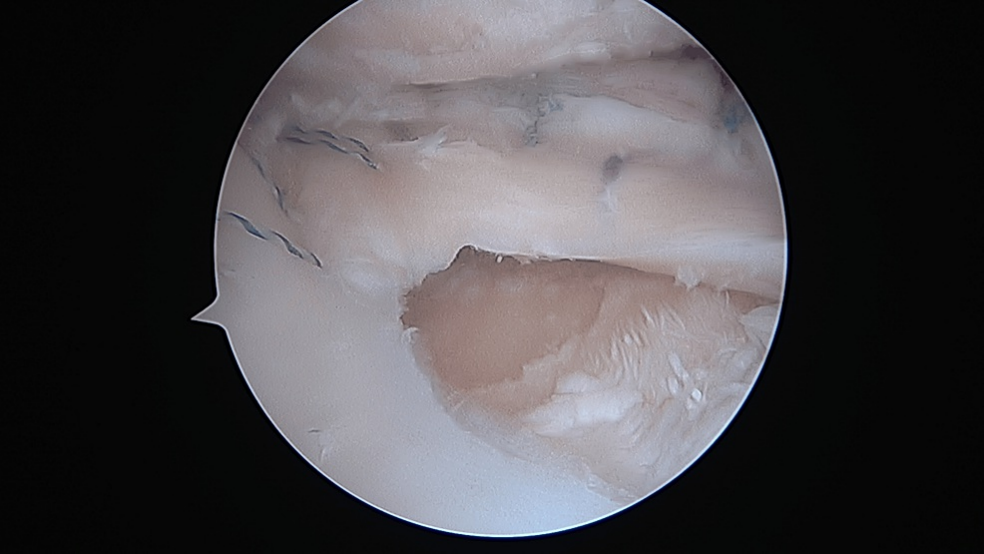
The suture of a meniscal allograft to peripheral capsule with inside, outside, and all-inside techniques

The allogenic meniscal graft was sutured to the peripheral capsule using all-inside, inside-out, and outside-in techniques (Figure 3).

OAT

The size of cartilage defects of less than 4.0 cm^2^ was indicated. Autologous osteochondral plugs (8.5 mm in diameter, 10-15 mm in length) were harvested from the non-weight-bearing area of the lateral femoral condyle (LFC) using an osteochondral autologous transfer system (Mosaicplasty®, Smith & Nephew, Andover, MA) and implanted in cartilage defects of the lateral FT condyles and patella. In one case, the allogenic tibial plateau bone with meniscus supplied by the Tissue Bank was prepared as the osteochondral plug and implanted in the LTP cartilage defect.

ACI

The size of cartilage defects of 4.0 cm^2^ or more was indicated. At the first two-staged operation, approximately 400 mg of cartilage slices were harvested from a non-weight-bearing area of the LFC via arthroscopy. These slices were sent to a Good Gene, Cellular, and Tissue-based Products Manufacturing Practice (GCTP) manufacturing facility [Japan Tissue Engineering (J-TEC) Co. Ltd., Aichi, Japan]. The cartilage tissue was subjected to enzymatic digestion. The chondrocytes were isolated, embedded in an atelocollagen gel, and cultured for four weeks in a three-dimensional manner to form the matrix-associated ACI product (JACC®, J-TEC Co. Ltd.). At the second operation, four weeks after the first, the chondral lesion was debrided from the intact surrounding cartilage until the subchondral bone was exposed via a medial or lateral parapatellar arthrotomy under tourniquet control. JACC®s were implanted into the cartilage defects of the femoral condyles, femoral trochlear, and patella beneath the sutured autologous periosteal flap from the proximal medial tibia. The joint capsule, retinaculum, and skin were sutured in separate layers.

Postoperative rehabilitation

The knee joint was immobilized at a slightly flexed knee position with a soft knee brace for one week after MAT implantation. Following this, knee joint exercise was initiated two weeks later, and partial and full weight-bearing walking was commenced at four and six weeks at our hospital, respectively. Light jogging was started six months postoperatively, and sports activities were started one year later. In the case of MAT with concomitant ACL-R, the rehabilitation protocol was modified according to that of ACL-R. A knee brace with a limited full extension was applied for three months after surgery. In the case of concomitant ACI, partial and full weight-bearing was delayed until six and eight weeks after ACI implantation.

Evaluation items

The effectiveness was evaluated clinically using the Lysholm knee scoring scale (LKSS) and Japanese Orthopaedic Association (JOA) knee score preoperatively, 12 and 24 months after the implantation. The standard arthrometer also assessed the knee joints' range of motion (ROM) during the visit.

Radiographic evaluations

The femorotibial angle (FTA) and width of the lateral FT joint space (LJSW) on the standing X-ray films of the knee joint were checked preoperatively, and one, 12, and 24 months after the implantation.

MRI evaluation of allogenic meniscal coronal extrusion

A conventional MRI of the transplanted fully-extended knee joint in a non-weight-bearing, supine position was performed one, 12, and 24 months after the implantation using 3.0-T MRI (Signa HDx 3.0T, GE Healthcare Milwaukee, WI). At serial proton-density or T2-weighted MRI images, maximum lateral meniscal extrusion (LME) was defined as the distance from the LFC or LTP to the meniscus's outer edge, resulting in a femoral and tibial extrusion distance [20]. LME data are presented as absolute values and as a relative percentage of extrusion (RPE), deﬁned as the percentage of the width of an extruded meniscus compared with the entire meniscal width [20].

The safety was assessed based on the incidence of adverse events determined by physical examination, MRI, and arthroscopically as needed; the adverse events were defined as undesired or unintended signs (e.g., abnormal laboratory test values), symptoms, or illnesses with or without a causal relationship to the MAT.

Statistical analysis

BellCurve for Excel (version 4.04) (Social Survey Research Information Co., Ltd., Tokyo, Japan) was used for statistical analyses. Demographic measurement data are presented as the mean, standard deviation (SD), and standard error (SE). The preoperative LKSS and JOA scores and those at 12 and 24 months after the implantation were compared among different time points before and after implantation using the Friedman test and Scheffe's multiple comparisons. FTA, LJSW, LME, and RPE were also compared preoperatively and/or one, 12, and 24 months after the implantation. A case with conversion to total knee arthroplasty (TKA) was not considered for imputation. Analytic data are presented as the median, top 25th percentile (first quartile), bottom 25th percentile (third quartile), minimum, maximum, and p-value. A p-value less than 0.05 was considered statistically significant.

## Results

Evaluation of clinical scores

In eight cases (except for one with TKA conversion), the median LKSS and JOA scores significantly improved from 70 points (first quartile-third quartile: 68.3-76.3 points) preoperatively to 85.5 (81.5-96) at 12 months, 86.5 (83.5-90.5) at 24 months (p=0.0013) and from 35 (33.8-45) to 85 (83.8-90), and 87.5 (85-90) (p<0.001), respectively (Table 4). The median ROM improved from 120º (113.8-127.5º) preoperatively to 127.5º (123.8-130º) at two years postoperatively, but it was not statistically significant (p=0.36) (Table 4).

**Table 4:** Clinical evaluation ROM: range of motion; LKSS: Lysholm knee scoring scale; JOA: Japan Orthopaedic Association Knee Score; Preop: preoperatively; 12 mos.: 12 months postoperatively; 24 mos.: 24 months postoperatively

Evaluation items	Evaluation period			Friedman test	Multiple comparison (Scheffe), p-value	
	Preop.	12 mos.	24 mos.	P-value	Preop. vs. 12 mos.	Preop. vs. 24 mos.
LKSS, point						
Median	70	85.5	86.5	0.0013	0.0046	0.011
1st-3rd quartile	68.3-76.3	81.5-96	83.5-90.5			
Min/max	21/80	65/100	65/98			
JOA, point						
Median	35	85	87.5	<0.001	0.013	0.0024
1st-3rd quartile	33.8-45	83.8-90	85-90			
Min/max	25/45	50/95	80/95			
ROM, degree						
Median	120	117.5	127.5	0.36	0.66	0.88
1st-3rd quartile	113.8-127.5	103.8-125	123.8-130			
Min/max	70/145	95/135	105/135			

Radiographic evaluation

In eight cases (except for one with TKA conversion), the change in median FTA and LJSW was not statistically significant: 171.5º (171-174.3º) preoperatively to 173.5º (172-174.8º) at 24 months postoperatively (p=0.20) and from 4.1 mm (3.2-6.8 mm) to 4.1 mm (3.6-6.0 mm) (p=0.64), respectively (Table 5).

**Table 5:** Radiographic evaluation FTA: femorotibial angle; LJSW: lateral joint space width; Preop.: preoperatively; 1 mo.: 1 month postoperatively; 12 mos.: 12 months postoperatively; 24 mos.: 24 months postoperatively

Evaluation items	Evaluation period				Friedman test	Multiple comparison (Scheffe), p-value	
	Preop.	1 mo.	12 mos.	24 mos.	P-value	Preop. vs. 12 mos.	Preop. vs. 24 mos.
FTA, degree							
Median	171.5	173.8	175.3	173.5	0.2	0.27	0.98
1st-3rd quartile	171-174.3	173-175.1	172.1-174.4	172-174.8			
Min/max	170.5/177	172/176	166/178	170/177.3			
LJSW, mm							
Median	4.1	4.56	4.8	4.1	0.64	0.81	0.99
1st-3rd quartile	3.2-6.8	2.7-4.8	4.0-5.7	3.6-6.0			
Min/max	2.0/7.3	2.6/5.2	2.8/8.7	2.3/8.3			

MRI evaluation of allogenic meniscal coronal extrusion

**Figure 4:**
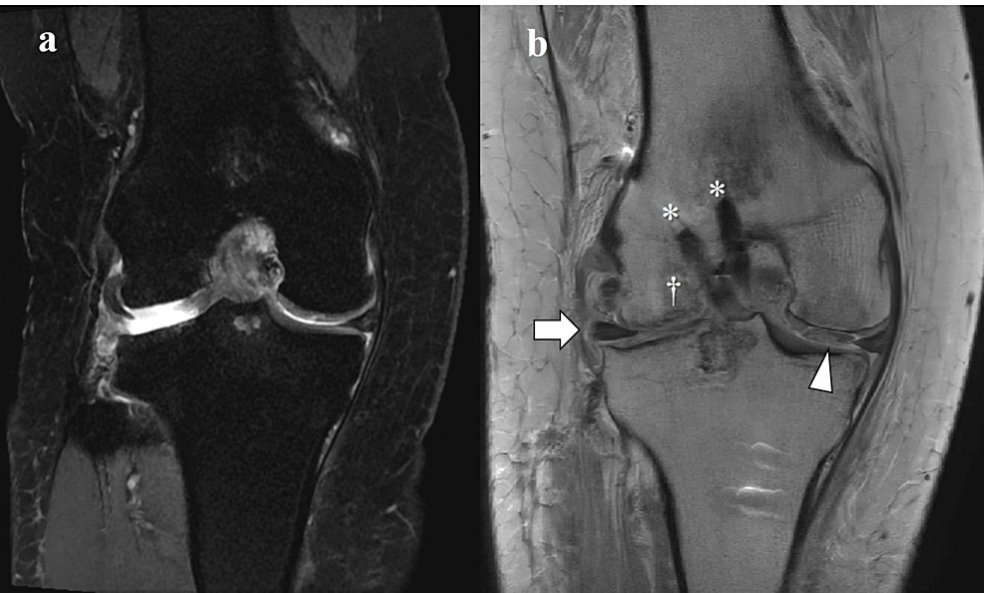
Coronal views of MRI (Case #4) - image 1 a. Preoperative proton density-weighted image (TR2427, TE 36.63, FOV:160). b. T2-weighted image (TR6000, TE 8.75, FOV:150) 1 month postoperatively *: Reconstructed anterior cruciate ligaments. †: Osteochondral autograft. White arrow: transplanted meniscal allograft. Arrowheads: autologous chondrocyte transplantation MRI: magnetic resonance imaging

**Figure 5:**
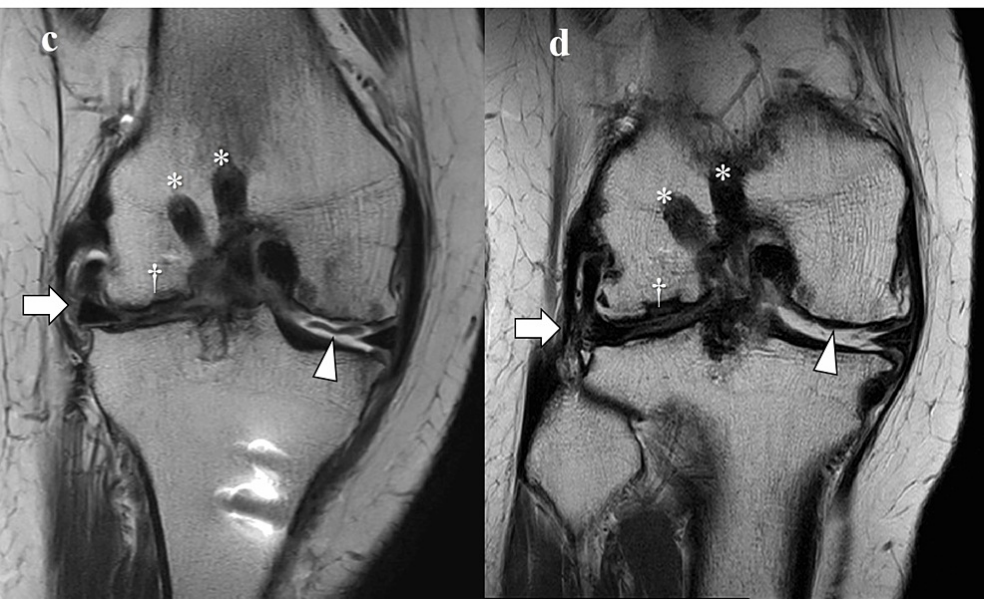
Coronal views of MRI (Case #4) - image 2 c. T2-weighted image (TR6000, TE 8.68, FOV:140) 12 months postoperatively. d. Proton density-weighted image (TR2400, TE 55.00, FOV:160) 24 months postoperatively *: Reconstructed anterior cruciate ligaments. †: Osteochondral autograft. White arrow: transplanted meniscal allograft. Arrowheads: autologous chondrocyte transplantation MRI: magnetic resonance imaging

LMEs increased by 3.0 mm (first quartile-third quartile: 2.4-3.9 mm) at one month postoperatively and remained unchanged until two years postoperatively [4.8 (3.6-5.0) at 12 months and 4.9 (3.9-5.0) at 24 months (p=0.079)] (Table 6; Figures 4, 5). RPE gradually increased, but not significantly (p=0.053) (Table 6). The proportion of the knees with major (≥3 mm) extrusion was 75% (six of eight cases) two years postoperatively.

**Table 6:** MRI evaluation of allogenic meniscal coronal extrusion LME: maximum lateral meniscus extrusion; MRI: magnetic resonance imaging; RPE: relative percentage of extrusion of lateral meniscus; Preop.: preoperatively; 1 mo.: 1 month postoperatively; 12 mos.: 12 months postoperatively; 24 mos.: 24 months postoperatively

Evaluation items	Evaluation period			Friedman test	Multiple comparison (Scheffe), p-value	
	1 mo.	12 mos.	24 mos.	P-value	1 mo. vs. 12 mos.	1 mo. vs. 24 mos.
LME, mm						
Median	3.0	4.8	4.9	0.079	0.2	0.12
1st-3rd quartile	2.4-3.9	3.6-5.0	3.9-5.0			
Min/max	0/6.3	2.6/5.9	2.5/5.9			
RPE, %						
Median	26.4	45.6	61.8	0.053	0.13	0.1
1st-3rd quartile	18.9-34.5	38.7-60.4	28.2-71.8			
Min/max	0/100	10.3/100	25.5/100			

**Figure 6:**
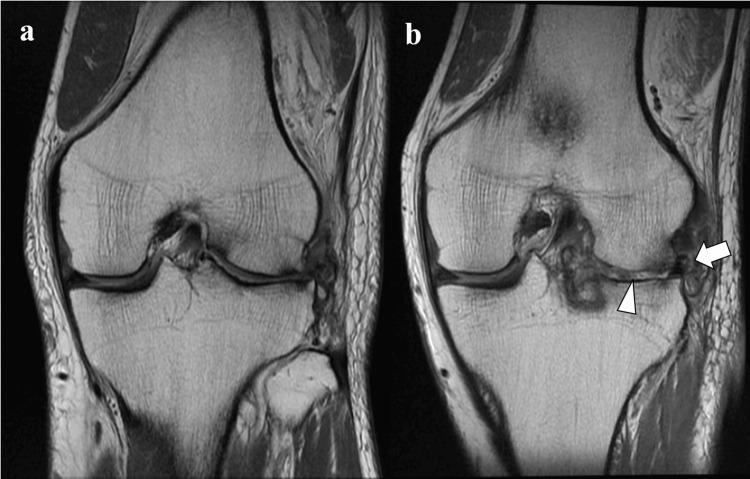
Coronal views of MRI (Case #8) a. Preoperative proton density-weighted image (TR2094, TE 30.46, FOV:160). b. Proton density-weighted image (TR2566, TE 22.87, FOV:160) 12 months postoperatively White arrow: meniscal allograft extrusion. Arrowhead: delaminated autologous chondrocyte transplantation MRI: magnetic resonance imaging

The treatment failed in one case [a 67-year-old male (case # 8)], who underwent MAT and simultaneous ACI for a large cartilage defect (10 cm^2^) in the LFC and femoral trochlear, resulting in no clinical improvement (preoperative LKS and JOA scores of 60 and 25) with the progression of the valgus knee from 172º to 168º in FTA and LME in MRI (Figure 6), and had to undergo TKA at 18 months postoperatively.

Blood exam showed neither infection nor abnormality as complications, except for lateral meniscal tear (three cases), contracture (two cases), and patellar instability (one case). Additional operations of meniscal sutures, arthroscopic arthrolysis, and medial patellofemoral ligament reconstruction concomitant with the anteromedial transfer of the tibial tuberosity were performed for each patient.

## Discussion

This pilot study constitutes the first comprehensive case series of MAT concomitant with OAT and/or ACI for symptomatic lateral meniscus-deficient knees in Japan. We found that MAT with cartilage repair provided significant clinical improvement in lateral meniscus-deficient knees by a median of 16.5 points in LKS and 52.5 points in JOA score at two years of follow-up. However, these procedures also led to unfavorable knee joint situations postoperatively in half of the cases. Hence, it is suggested that this technique should be only adopted in select cases and performed by only a handful of highly experienced surgeons.

In the context of restriction to using allogenic allograft in Japan, Nishitani et al. reported 10 cases of isolated OAT for lateral meniscus-deficient knees with cartilage defect over 73.8 ±42.5 months of follow-up, resulting in significant clinical improvement in seven cases (70%) except for one that involved revised TKA and two progressions of valgus malalignment [21]. Their study included relatively younger patients (31.7 ±19.7 years) with smaller cartilage defects (3.5 ±1.7 cm^2^) than those in the current study. Their results suggest that MAT with the goal of additive chondroprotective effects might be necessitated for symptomatic lateral meniscus-deficient knees with larger cartilage defects.

Distal femoral varus osteotomy (DFO) might be another alternative for treating symptomatic lateral meniscus-deficient knees. In a systematic review, Rao et al. recommended DFO for physically young patients, with valgus deformity of >12-15º, joint-line obliquity >10º, flexion of at least 90º, and <15º flexion contracture [5]. With the conversion to TKA as the endpoint, mean survivorship at 10-year follow-up was 64-87% [5]. Although the long-term results of MAT and cartilage repair in the present study remain unclear, the current cases were excluded from the above indication of DFO.

Since the first report by Gersoff in 2002 [14], there have been a few reports of combined MAT and cartilage repair. In 2007, Bhosale et al. reported eight cases of meniscus-deficient knees (medial/lateral: 2/6) with cartilage defects (size: 9.7 ±3.7 cm^2^) treated by MAT combined with ACI with one-year follow-up, resulting in a mean LKSS from 49 preoperatively to 66 at one year (average increase of 16.4 points) [22]. Also, Farr et al. reported 29 cases with deficient menisci (medial/lateral: 21/8) and cartilage defects (size: 6.36 ±3.26 cm^2^) treated by the same combined procedures, resulting in an improvement in LKSS from 57.7 ±16.2 to 77.7 ±19.3 with at least two years of follow up. However, they included concomitant high tibial osteotomies (six cases) and tibial tubercle osteotomies (two cases) [23]. A prospective evaluation of 31 patients with cartilage defects (mean: 4.68 cm^2^) by Rue et al. demonstrated improvement in LKSS from 48.7 ±16.4 to 74.0 ±17.7 and satisfaction with outcomes in 76% of the participants for MAT concomitant with ACI (80% of participants) and osteochondral allograft (36%) [24]. Harris et al. systematically reviewed six studies, including these reports in 2010, demonstrating that combined MAT and articular surgeries improved clinical outcomes in a manner comparable to that for an isolated procedure of MAT or articular surgeries [25]. However, their combined articular surgeries, such as OAT, ACI, arthroscopic debridement, and bone mallow stimulation, were miscellaneous in nature. Treatment failed in 12% of the cases, 85% of which were isolated-MAT failures [25]. Ogura et al. demonstrated a 75% survival rate for combined MAT and ACI at both five and 10 years in 17 patients (18 knees; mean age: 31.7 years; cartilage defect size - mean, 7.6 cm^2^/knee) over a mean 7.9-year follow-up [15]. This report might support the notion that MAT combined with ACI might promise a mid- and long-term improvement in meniscus-deficient knees with cartilage defects.

The current study also demonstrated that the transplanted allogenic lateral meniscus extruded by ≥3 mm and RPE gradually increased two years after implantation. A systematic review of 21 studies by Lee showed that the overall mean absolute extrusion, RME, and proportion of the knees with major (≥3 mm) extrusion were 3.15 mm (medial/lateral: 3.26/3.01 mm), 32.79% (32.69%/28.81%), and 53% (61%/39%), respectively, concluding that graft extrusion after MAT could not be wholly avoided [26]. Since MAT does not reconstruct the coronary ligament, meniscal extrusion can be attributed to the loosening of Meniscocapsular attachments [27]. Lee et al. demonstrated that extrusions remained unchanged at one year, four to six years, and >8 years after lateral MAT of 27 cases over 10 years [27]. Son et al. also observed no significant differences in meniscal width, thickness, and intrameniscal intensity in lateral meniscal allografts for the mid-term follow-up (three to seven years) [28]. Both studies showed that clinical improvements were maintained independently from meniscal extrusions and degenerative morphogenic changes in the mid- and long-term [27,28]. However, their indications for MAT included articular chondral wear classified as Outerbridge grade II or less or articular chondral grade III or IV but with localized wear in the lateral compartment. Because this study shows that 75% of cases had major (≥3mm) extrusion, and RPE gradually increased at the final follow-up, it might be a concern that the long-term meniscal extrusion worsens the chondroprotective effect in cartilage-repaired knees.

The procedural success related to adding alignment correction to MAT and/or cartilage repair is controversial. Stone et al. reported no improvement in patients with Outerbridge III or IV cartilage degeneration treated using MAT combined with alignment correction, suggesting that axial malalignment <7º did not affect survival in MAT [29]. They addressed osteotomy in case of malalignment of >7º. The International Meniscus Reconstruction Experts Forum (IMREF) has recommended that realignment osteotomy be combined with MAT when the mechanical axis falls within the affected compartment [30]. Although the FTA in current cases ranged from 171º to 177º, additional alignment correction might be necessary for MAT's durability and cartilage repair.

This pilot study has a few limitations, such as the fact that it included very few lateral meniscus-deficient knees, and involved various cases, short-term follow-up, and non-randomized analysis. Further studies are required to address these issues and gain deeper insights into the effectiveness and safety of MAT combined with cartilage repair for symptomatic meniscus-deficient knees. More data on MAT performed simultaneously with cartilage repair should be gathered to establish evidence of the efficacy and safety of the procedures in Japan.

## Conclusions

Based on our findings, MAT concomitant with cartilage repair showed good clinical outcomes two years postoperatively. However, three-quarters of meniscus allografts led to large extrusions, and half of the cases required additional surgery. Hence, this technique should be adopted in select cases only and performed by only a handful of highly experienced surgeons. Further research is needed to establish the chondroprotective effect of MAT and cartilage repair on symptomatic lateral meniscus-deficient knees.
